# Volumetric lung nodule segmentation using adaptive ROI with multi-view residual learning

**DOI:** 10.1038/s41598-020-69817-y

**Published:** 2020-07-30

**Authors:** Muhammad Usman, Byoung-Dai Lee, Shi-Sub Byon, Sung-Hyun Kim, Byung-il Lee, Yeong-Gil Shin

**Affiliations:** 1 Department of Computer Science and Engineering, Seoul National University, 08826 Seoul, South Korea; 2 Center for Artificial Intelligence in Medicine and Imaging, HealthHub Co. Ltd., Seoul, 06524 South Korea; 3 School of Computer Science and Engineering, Kyonggi University, Suwon, 16227 South Korea

**Keywords:** Information technology, Computer science

## Abstract

Accurate quantification of pulmonary nodules can greatly assist the early diagnosis of lung cancer, enhancing patient survival possibilities. A number of nodule segmentation techniques, which either rely on a radiologist-provided 3-D volume of interest (VOI) or use the constant region of interests (ROIs) for all the slices, are proposed; however, these techniques can only investigate the presence of nodule voxels within the given VOI. Such approaches restrain the solutions to freely investigate the nodule presence outside the given VOI and also include the redundant structures (non-nodule) into VOI, which limits the segmentation accuracy. In this work, a novel semi-automated approach for 3-D segmentation of lung nodule in computerized tomography scans, has been proposed. The technique is segregated into two stages. In the first stage, a 2-D ROI containing the nodule is provided as an input to perform a patch-wise exploration along the axial axis using a novel adaptive ROI algorithm. This strategy enables the dynamic selection of the ROI in the surrounding slices to investigate the presence of nodules using a Deep Residual U-Net architecture. This stage provides the initial estimation of the nodule utilized to extract the VOI. In the second stage, the extracted VOI is further explored along the coronal and sagittal axes, in patchwise fashion, with Residual U-Nets. All the estimated masks are then fed into a consensus module to produce a final volumetric segmentation of the nodule. The algorithm is rigorously evaluated on LIDC–IDRI dataset, which is the largest publicly available dataset. The proposed approach achieved the average dice score of 87.5%, which is significantly higher than the existing state-of-the-art techniques.

## Introduction

Lung cancer is one of the most severe and highly-prevalent cancers and is the leading cause of cancer deaths worldwide^1^. It has been forecasted to be one of the greatest single cause of mortality among the European population in 2019^2^. Early diagnosis of lung cancer is crucial to enable possible life-saving interventions^3^, which relies on accurate quantification of pulmonary nodule; albeit pulmonary nodules can be associated with several diseases, their recurrent diagnosis is lung cancer. The continuous monitoring of lung nodule volume is vital to estimate the malignancy and to better forecast, the probability of lung cancer^4,5^. For calculation of volume, the nodule is first segmented, while the manual segmentation of nodule is a tedious and time-consuming task which also introduces the inter and intra-observer variabilities^6^.

**Figure 1:**
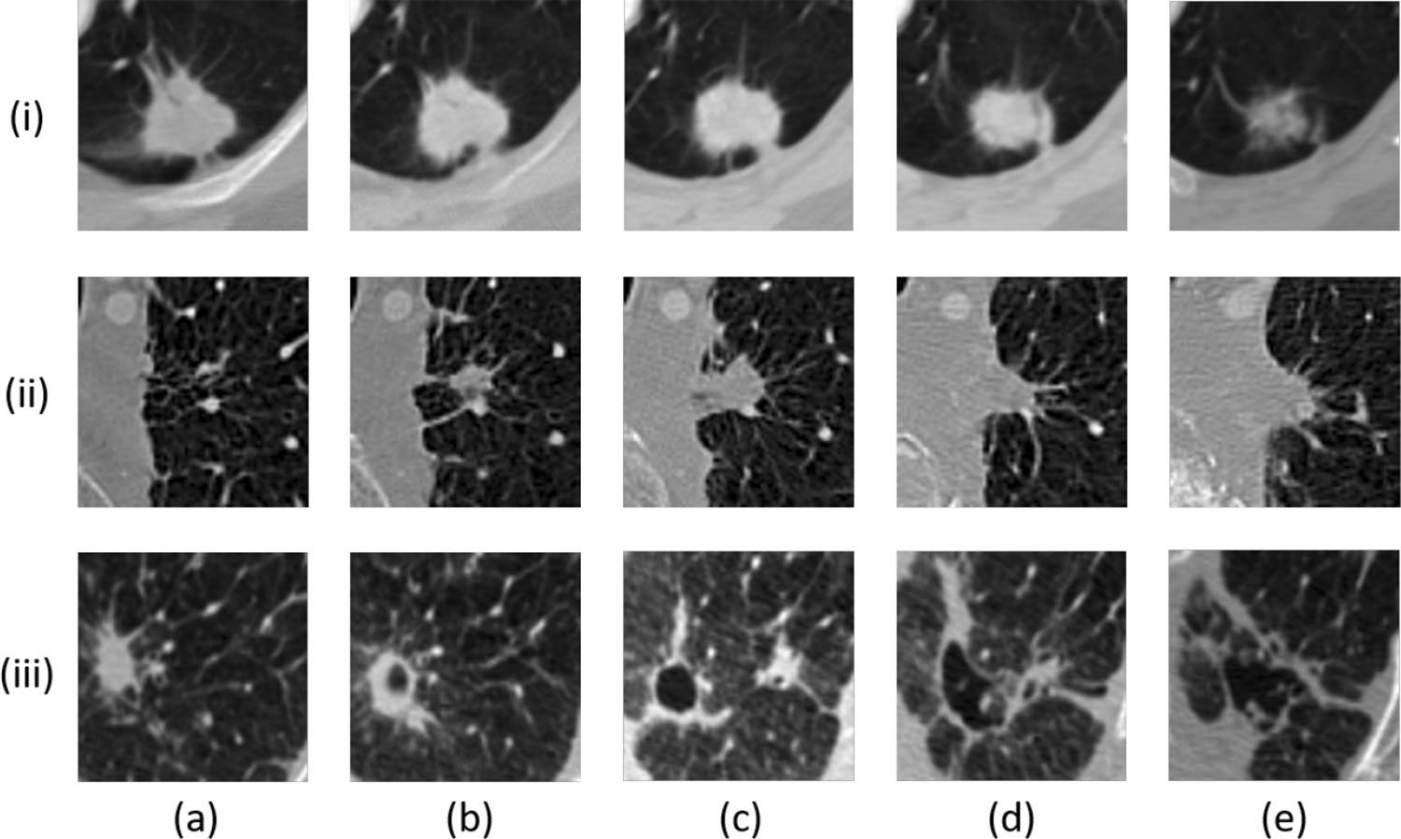
Multiple visual appearances of the pulmonary nodule are shown. The intra-nodule variation in slices of axial view is depicted from column (**a**) to (**e**), and the inter-nodule difference is presented from rows (i) to (iii).

Computer-aided diagnosis (CAD) systems have huge potential to overcome the challenges faced during manual segmentation of pulmonary nodules and can remarkably enhance the productivity of radiologists. Therefore, several automatic nodule segmentation techniques have been proposed to facilitate radiologists, including advanced deep learning and classical image processing based techniques^7^. All existing techniques require a 3-D volume of interest (VOI) as an input to precisely estimate the shape of a nodule^8–10^, this VOI can either be an output of nodule detection module or can be provided by the radiologist. However, despite of having VOI, the shape variations within a nodule and the visual similarity between a nodule and its surroundings (i.e., non-nodule tissue) act as barriers toward the development of a highly accurate and robust nodule segmentation solution. Figure 1 illustrates intra-nodule variations in slices of axial view and also inter-nodule variations, wherein the diversity between the shapes of different nodules and multiple axial views of a single nodule are observable.

Volumetric segmentation of pulmonary nodule can be performed either by using 3-D VOI-based segmentation technique or by performing 2-D region of interest (ROI) based segmentation in the patch-wise fashion. However, there are several challenges associated with 3-D VOI-based segmentation, such as demand for a large amount of training data and higher computation cost. On the other hand, 2-D ROI-based segmentation is significantly faster, required less training data and computational power, which makes it a popular choice. Moreover, recent studies^7,11^ have also demonstrated that through patch-wise investigation, 2-D segmentation scheme can be efficiently exploited to estimate the 3-D volumetric shape of a lung nodule.

In this work, we propose a novel approach for volumetric segmentation of pulmonary nodules by taking only the 2-D ROI input from the radiologist. The solution first explores the presence of a nodule within the provided ROI by employing a Deep Residual U-Net and then extends the search into surrounding slices (i.e., in both directions). To investigate possible penetration of the nodule within adjacent slices, we introduce the concept of adaptive ROI (A-ROI) that allows the solution to dynamically change the position and size of ROI while searching into other slices. To the best of our knowledge, such A-ROI algorithm has never been proposed for the optimization of volumetic segmentation in any medical imaging modality. The application of this A-ROI algorithm along the axial plane provides an initial estimation of the nodule shape, which is leveraged to extract a 3-D VOI from the scan automatically. This VOI is further utilized to create coronal and sagittal views of the nodule, and slices of both are investigated with two Deep Residual U-Nets. Finally, three estimated segmentation masks (i.e., for the axial, coronal, and sagittal views) are fed into a consensus module to build a final segmentation mask. To validate the performance of the proposed method, an extensive set of experiments has been conducted on LIDC–IDRI dataset^12^, which is the largest publicly available dataset. The results suggest that the approach is robust and significantly improves the performance in terms of dice score as compared to the previous state-of-the-art techniques.

## Related work

An accurate assessment of the lung nodule is required to investigate its malignancy and possibility of lung cancer. Due to the exceptional importance of nodule segmentation, several efforts have been made to develop a highly accurate and robust automatic nodule segmentation system that may assist radiologists. These efforts can be distinguished into two categories: deep learning-based techniques and classical image processing-based techniques^7,11^. In this section, we incorporate a brief review of recently proposed techniques from each category.

Jamshid et al.^13^ proposed an algorithm that utilized two-region growing techniques (i.e., fuzzy connectivity and contrast-based region growing) to perform nodule segmentation. The region growing is operated within a volumetric mask that is created by first applying a local adaptive segmentation algorithm to identify the foreground and background regions within a specified window size. The algorithm performed well for a separated nodule but failed to segment the attached nodules. Stefano et al.^14^ proposed a user interactive algorithm that adopts geodesic influence zones in a multi-threshold image representation to allow the achievement of fusion–segregation criteria based on both gray-level similarity and objects shape. The same author extended this work^15^ by removing the user interaction component and performing corrections according to 3-D local shape analysis. The correction procedure refined an initial nodule segmentation to split possible vessels from the nodule segmentation itself.

Threshold and morphological techniques were adopted in Elmar et al.^16^ to eliminate the background and other surrounding information from the provided ROI. Then, a support vector machine (SVM) was employed to classify each pixel in the detected space. Similarly, Wang et al.^17^ tried to segment solitary pulmonary nodules in digital radiography (DR) images by incorporating a sequential filter to construct new representations of the weight and probability matrices. However, this method is limited to DR images, which constrains the application for CT images. Additionally, Julip et al.^18^ segmented ground-glass nodules (GGN) in chest CT images using an asymmetric multi-phase deformable model. However, this technique lacks the robustness to address segmentation requirements for other nodule types.

Shakibapour et al.^9^ employed the notion of optimally clustering a set of feature vectors comprising intensity and shape-related features in a given feature data space extracted from a predicted nodule. The size is obtained by measuring the volume of a segmented nodule via an ellipsoid approximation using the equivalent diameters of the segmented regions in a 2.5-D representation; thus, the uncertainty persists into the final results. Shakir et al.^19^ proposed the voxel intensity-based segmentation scheme that incorporates mean intensity-based thresholding in the Geodesic Active contour model in level sets. This work was validated on limited set of scans, so the robustness of the proposed technique is dubious.

**Figure 2:**
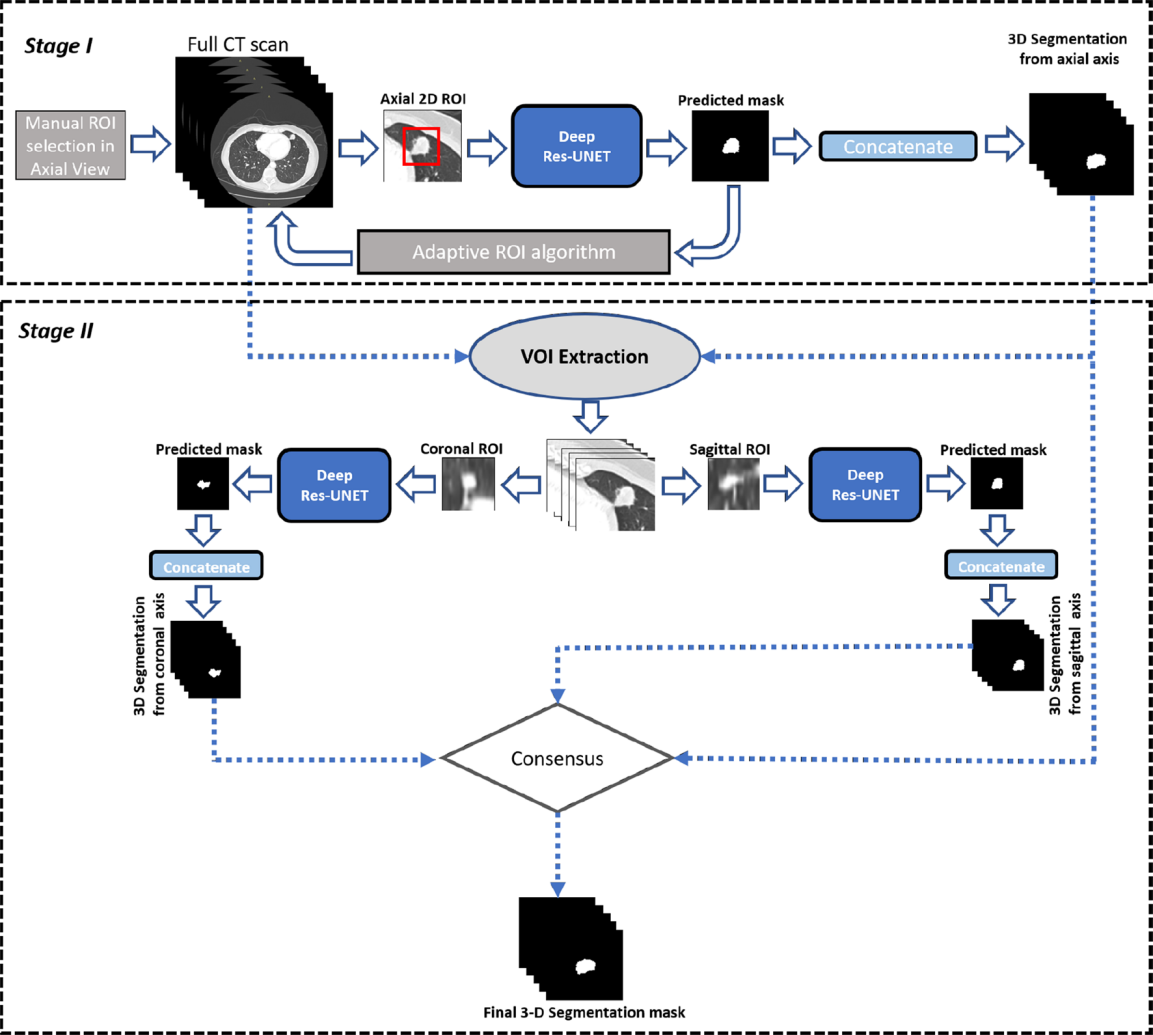
An illustration of the stages of the proposed method. At stage I, a manual ROI along the axial axis is provided by the user, and a Deep Residual U-Net along with the adaptive ROI algorithm is employed to extract the volume of interest (VOI). After getting the VOI during stage II, a patch-wise segmentation of the nodule is performed along the coronal and sagittal axes. Eventually, a consensus module is employed on all estimated segmentation masks to obtain the final 3-D nodule segmentation mask.

Despite extensive research, classical image processing techniques were unable to provide sufficiently robust and accurate volumetric nodule segmentation. On the other hand, recent advancements in deep learning (DL) have revolutionized image enhancement^20^ and segmentation-related applications^21,22^, including lung nodule segmentation tasks^23^. Especially the introduction of the U-Net architecture^24^, for segmentation in medical images, has remarkably enhanced the performance for these tasks. Subsequently, many efforts have been made to leverage DL-based techniques for lung nodule segmentation, such as Wang et al.^8^ developed the Central Focused Convolutional Neural Network (CF-CNN) that takes a volumetric patch around a selected voxel as the input and returns the segmentation of nodule. The same authors in^25^ presented a multi-view CNN for lung nodule segmentation, which takes the axial, coronal and sagittal view around a given voxel of a nodule as input and provides nodule segmentation. The method of patch (axial, coronal, and sagittal) extraction around the given nodule is kept fixed for all nodules which may lead to compromised segmentation if the nodule is larger than the extracted patch size.

Guofeng et al.^26^ improved the performance of U-Net for nodule segmentation by including skipped connections within the encoder and decoder paths. The study reports an enhanced performance of U-Net but lacks from 3-D volumetric analysis. Similarly, Amorim et al.^27^ modified the U-Net architecture to investigate the presence of nodules through a patch-wise approach. They manually extracted a VOI containing the nodule and performed 2-D segmentation along each axis, so a final segmentation is calculated by summing the three predicted masks. The VOI selection criterion was static and is fixed as a 128 $\times$ 128 $\times$ 12 window around the given voxel, which deprives the solution to achieve high accuracy on the large nodules. Hancock et al.^28^ presented an extension of the vanilla level set image segmentation method in which instead of being manually designed, the velocity function is learned from data via machine learning regression methods. They employed their segmentation scheme for lung nodule segmentation and reported slightly improved performance. A residual block based dual-path network in Liu et al.^10^ extracts local features and rich contextual information of lung nodules, which resulted in a significant performance enhancement. However, they also used a fixed VOI that refrains the free search of the nodule and subsequently reduces the performance.

In this work, we eliminate the downsides of the fixed VOI by introducing the adaptive 2-D ROI selection algorithm, which greatly assists the solution to exploit the power of deep learning. Afterwards, we utilize the Deep Residual U-Net^29^, which has shown excellent performance for other segmentation tasks but has never been employed for nodule segmentation. Finally, We investigate the automatically extracted VOI along the coronal and sagittal axes to determine an accurate segmentation of a lung nodule.

## Proposed method

Our method consists of two stages, as described in Fig. 2. In the first stage, we estimate the nodule 3-D shape along the axial axis to extract the VOI. In the second stage, we utilize the extracted VOI to further perform 2-D patch-wise investigation along the sagittal and coronal axes. Finally, we use the consensus module to calculate the 3D segmentation of the nodule. Details of each stage are described below.

### Stage I

The 2-D ROI containing a nodule is manually provided by the radiologist and may be selected from any portion of the nodule (i.e., from any slice). This ROI is then processed by the Deep Residual U-Net architecture to obtain a 2-D segmentation of the nodule, which is next forwarded into the A-ROI algorithm; the algorithm utilizes the position of nodule within the current ROI to determine the size and position of the ROI for the subsequent slice. Finally, the volumetric segmentation mask of the nodule is built by concatenating all the 2-D segmentation masks.

Descriptions of the A-ROI algorithm and Deep Residual U-Net architecture are provided in the following sections.

#### Adaptive ROI algorithm

**Figure 3:**
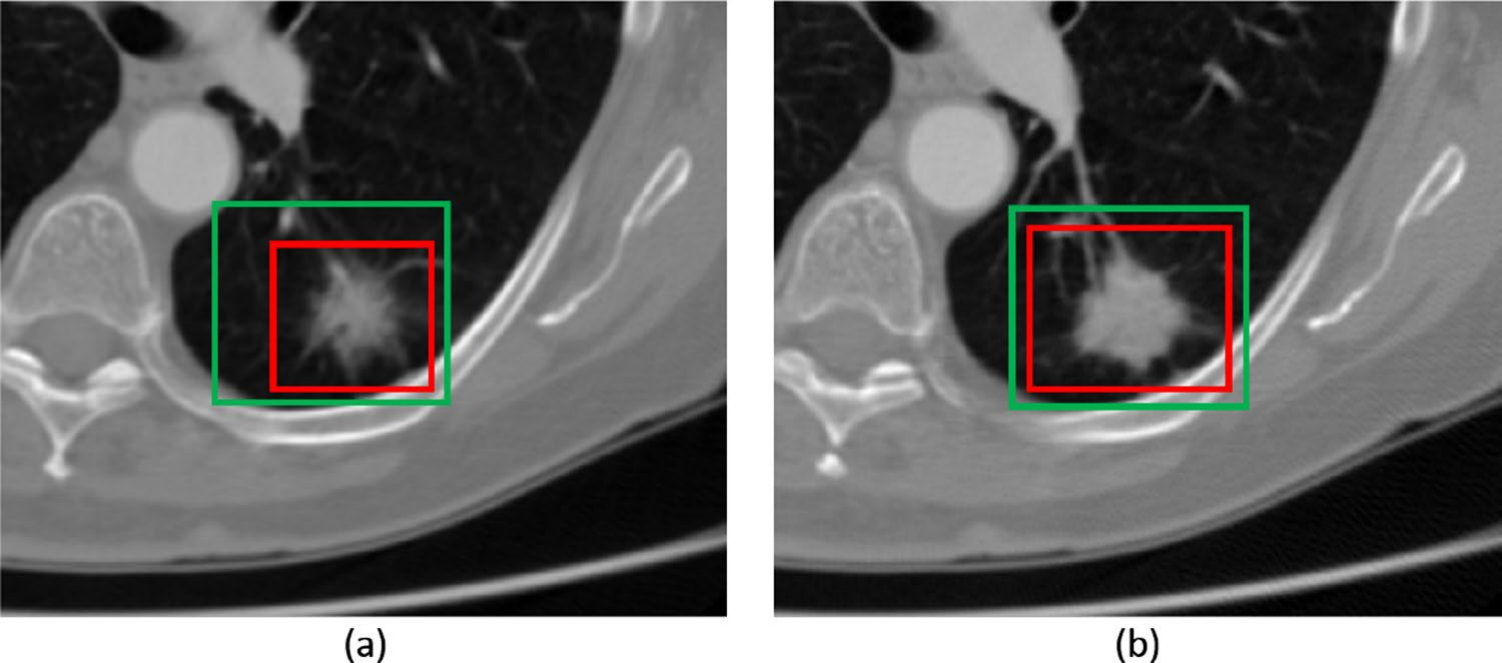
The change in position and shape of the ROI after employing our A-ROI algorithm, is presented. The current and next slice of VOI have been shown in figure (**a**,**b**), respectively. The red ROI demonstrates the change in size of ROI by A-ROI algorithm while the change in position of the ROI is depicted by green ROI.

The adaptive ROI algorithm dynamically selects the ROI for next slice to search for the presence of a nodule. The objectives of this algorithm include: to keep the ROI concentric to the predicted nodule mask by adjusting the position of the ROI and to maintain the ratio between the area of the nodule and ROI below a threshold level ($R_T$), by changing the the size of the ROI. Figure 3 illustrates the affect of A-ROI algorithm, current and next slices have been presented in (a) and (b), respectively. Red colour ROI demonstrates the change in the size of ROI by A-ROI algorithm and green colour ROI presents the change in the position of ROI from current to next slice.

**Figure 4:**
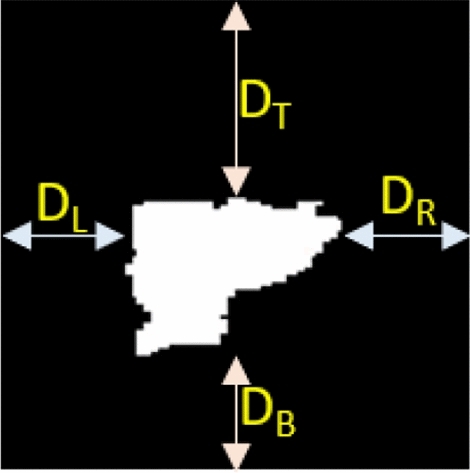
The margins on the four sides of the predicted mask are highlighted as $D_L, D_R, D_T,$ and $D_B$, for the left, right, top, and bottom margins of the predicted nodule segmentation, respectively.

Algorithm 1 describes the steps of the nodule penetration search into adjacent slices. First, nodule segmentation is performed on the manual ROI provided by the user. After obtaining a predicted segmentation of a nodule, the ROI position and size are adjusted by considering the margins identified in the predicted mask.

In Algorithm 1, $A_{N_i}$, $A_{ROI_i}$, and $A_{ROI_{i+1}}$ indicate the area of a nodule in the current ($i_{th}$) slice, the area of the ROI in the current ($i_{th}$) slice, and the suggested area of ROI in next ($(i\pm 1)_{th}$) slice, respectively. $D_L$, $D_R$, $D_T$, and $D_B$ are the left, right, top and bottom margins of the predicted mask, respectively, as shown in Fig. 4, while $\Delta D_X$ and $\Delta D_Y$ provide the differentials in the margins along the x and y axes, respectively. $X_1, X_2$ and $Y_1, Y_2$ are the beginning and ending coordinate points along the *x* and *y* axes of the current ROI, and $X'_1, X'_2$ and $Y'_1, Y'_2$ are the beginning and ending coordinate points along the *x* and *y* axes of the updated ROI in the next slice ($S_{i\pm 1}$), respectively.

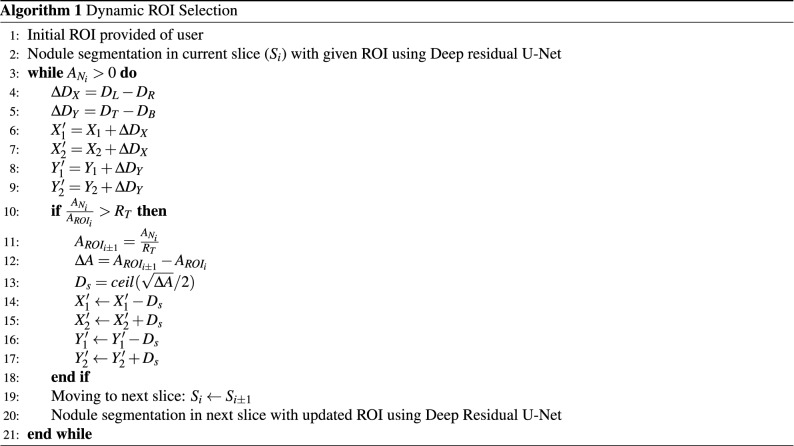



After determining the position of the next ROI, Algorithm 1 determines the optimal size of the ROI. The shape of the ROI remains square so that each side of the calculated ROI has the same length. The algorithm retains the ratio between $A_N$ and $A_{ROI}$, which is less than the constant value of the selected ratio threshold ($R_T$). The value of $R_{T}$ is crucial, as it determines the size of the ROI to be selected for the next slice, which is directly linked to the maximum possible movement of the nodule within two adjacent slices. The size of the ROI should be adequate to address the maximum possible displacement of the nodule that is directly proportional to the slice thickness (*ST*). Therefore,

1 $$\begin{aligned} R_T \propto \frac{1}{ST} . \end{aligned}$$

The optimum value of $R_T$ is determined experimentally by using validation data and is discussed in the “Results and discussion” section. Eventually, the A-ROI algorithm tends to retain the condition,

2 $$\begin{aligned} R_T \le \frac{A_N}{A_{ROI}} . \end{aligned}$$

Here, the minimum possible value of $A_{ROI}$ can be equal to $A_N$, so $R_T \in (0,1)$.

If the ratio of $A_{N_i}$ and $A_{ROI_i}$ becomes greater than $R_T$, then the algorithm calculates the differential ($\Delta A$) between the current area of the ROI ($A_{ROI_i}$) and the required area of the ROI for next slice ($A_{ROI_{i+1}}$). Then, $\Delta A$ is utilized for updating the coordinates of the ROI to obtain the required size.

In Fig. 3, the ROI within the current slice and the estimated ROI (calculated using the A-ROI algorithm) for the next slice are shown in red and green, respectively. The change in the position of the ROI is presented in Fig. 3a,b, the change of the size of the ROI is depicted.

**Figure 5:**
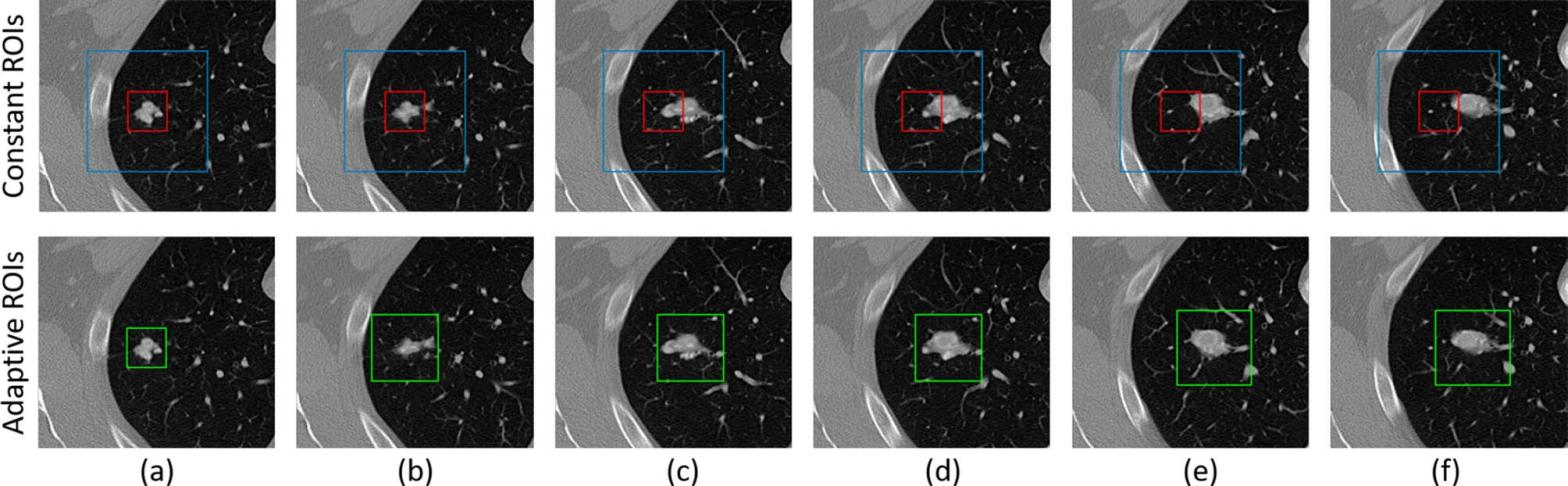
The constant and adaptive region of interests (ROIs) have been shown in a sequence of slices in which nodule is present. Blue and red boxes represent the constant ROIs, while green boxes depict the adaptive ROI.

The entire effect of the A-ROI algorithm is observed in Fig. 5, where two constant ROIs and an adaptive ROI are shown. The segmentation of the nodule starts from column (a) with the manual ROI and ends at column (f). The conventional ROIs (i.e., in red and blue) are the same in each slice while the adaptive ROIs, presented in green, feature different positions and sizes in each slice. In a conventional approach when the ROI remains close to the nodule in the initial slice (as shown in red), it fails to cover the nodule area present in the other slices. Then, a constant and larger ROI (shown in blue ) is required to incorporate the complete area of a nodule spanning all slices. This approach also adds redundant information into the ROIs, which impacts the performance of the segmentation model. On the other hand, our adaptive ROI strategy not only enables the user to select the ROI without the need to look into other slices, but also optimally chooses the ROIs for the remainder of the slices to facilitate improved performance of the segmentation model.

**Figure 6:**
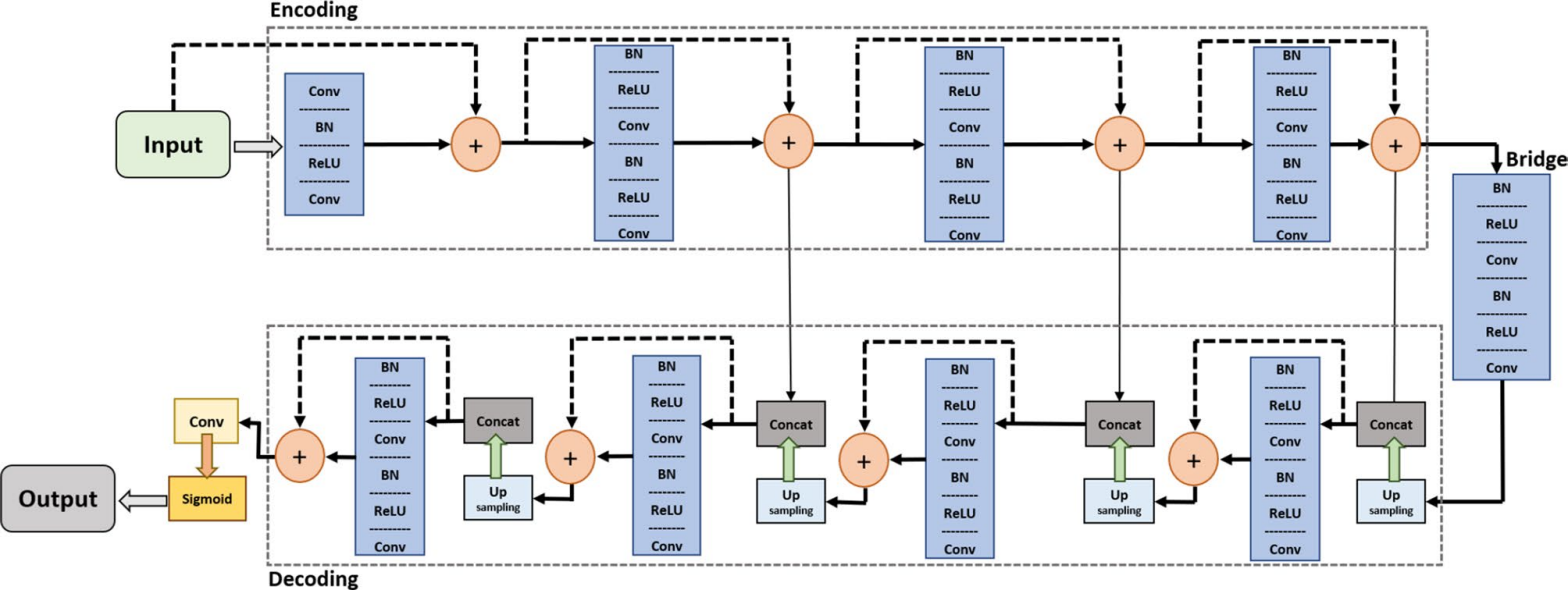
The architecture of Deep Residual U-Net, which is employed along the axial axis with the A-ROI algorithm to perform the lung nodule segmentation.

#### Deep Residual U-Net architecture

For nodule segmentation in the provided and calculated ROIs by A-ROI algorithm, we employ the Deep Residual U-Net architecture^29^. The network utilizes the residual learning in the U-Net architecture, which is state-of-the-art for segmentation. The involvement of residual units eases the training process, and the skip connections allow the flow of information without degradation from low levels to high levels of the network. This combination enables the network to learn the patterns with fewer parameters, which is significant for our application, and increases the performance on conventional U-Net architectures.

We leverage a 9-level architecture of the Deep Residual U-Net , as shown in Fig. 6. The network consists of three sections for encoding, a bridge, and decoding. The encoding section contracts the input image into a compact representation. The decoding section recovers the required information (i.e., the semantic segmentation) into a pixel-wise representation. The bridge connects these two sections. All sections of the network are built with residual units consisting of two $3 \times 3$ convolution blocks that include a batch normalization layer, a ReLU activation layer, and a convolutional layer. The identity mapping connects the input and output of the unit.

The encoding path has four residual units. In each unit, instead of using a pooling operation to down-sample the feature map size, a stride of 2 is applied to the first convolution block to reduce the feature map by half. Correspondingly, the decoding path also comprises four residual units. Preceding each unit, an up-sampling of the feature maps occurs from the lower level, and a concatenation of the feature maps is applied from the corresponding encoding path. After the final level of the decoding path, a $1\times 1$ convolution and sigmoid activation layer is used to project the multi-channel feature maps into the desired segmentation. The parameters and output sizes of each step are listed in Table 1.

**Table 1:** The network structure of the Deep Residual U-Net that performs patch-wise segmentation along the axial axis.

Unit level	Conv layer	Filter	Stride	Output size
**Input**				$128 \times 128 \times 1$
Level 1	Conv 1 Conv 2	$3 \times 3/64$ $3 \times 3/64$	1	$128 \times 128\times 64$ $128 \times 128 \times 64$
			1	
Level 2	Conv 3 Conv 4	$3 \times 3/128$ $3 \times 3/128$	2	$64 \times 64 \times 128$ $64 \times 64 \times 128$
			1	
Level 3	Conv 5 Conv 6	$3 \times 3/256$ $3 \times 3/256$	2	$32 \times 32 \times 256$ $32 \times 32 \times 256$
			1	
Level 4	Conv 7 Conv 8	$3 \times 3/512$ $3 \times 3/512$	2	$16 \times 16 \times 512$ $16 \times 16 \times 512$
			1	
Level 5	Conv 9 Conv 10	$3 \times 3/1024$ $3 \times 3/1024$	2	$8 \times 8 \times 1024$ $8 \times 8 \times 1024$
			1	
Level 6	Conv 11 Conv 12	$3 \times 3/512$ $3 \times 3/512$	1	$16 \times 16 \times 512$ $16 \times 16 \times 512$
			1	
Level 7	Conv 13 Conv 14	$3 \times 3/256$ $3 \times 3/256$	1	$32 \times 32 \times 256$ $32 \times 32 \times 256$
			1	
Level 8	Conv 15 Conv 16	$3 \times 3/128$ $3 \times 3/128$	1	$64 \times 64 \times 128$ $64 \times 64 \times 128$
			1	
Level 9	Conv 17 Conv 18	$3 \times 3/64$ $3 \times 3/64$	1	$128 \times 128 \times 64$ $128 \times 128 \times 64$
			1	
**Output**	Conv 19	$1 \times 1$	1	$128 \times 128 \times 1$

#### Loss function

Given a set of training images and the corresponding ground truth segmentations, ${I_i, s_i}$, we estimate the parameter W of the network, such that it produces accurate and robust nodule segmentation masks. This optimal value is achieved through minimizing the loss between the segmentations generated by $Net(I_i,W)$ and the ground truth $s_i$. We use the dice similarity coefficient (DSC)^30^ as the loss function,

3 $$\begin{aligned} \textit{L(W)} = \frac{1}{N}\sum _{i=1}^{N} \left[ 1 - \frac{2*Net(I_i;W)\cap s_i}{Net(I_i;W) \, {\cup } \, s_i}\right] \end{aligned}$$

where *N* is the number of training samples. We use stochastic gradient descent (SGD) to train our network.

### Stage II

The second stage of our approach is further designated into two phases. The first employs patch-wise nodule segmentation along the coronal and sagittal axes, and the second reconstructs the final 3-D segmentation mask of the nodule from all estimated nodule segmentations. These phases are detailed in the following.

#### Multi-view investigation

The VOI extracted from stage I is utilized to perform patch-wise nodule segmentation along the coronal and sagittal axes independently with two networks. By considering the fact that slice thickness is often greater than the voxels spacing in the x–y plane, we resize the coronal and sagittal patches to $128\times 64$. The segmentation is performed using a similar Deep Residual U-Net architecture as that used during stage I. However, owing to the smaller size of the images, the number of levels is reduced to seven (i.e., three encoding, one bridge, and three decoding units), which decreases the number of parameters in the network; this is shown in Table 2. The output of each model is resized to the original size of the ROI; original size refers to the size in which the ROI was extracted from scan. Finally, all the inferences are concatenated in the same manner as it was performed during stage I to reconstruct the 3D segmentation of a nodule.

**Table 2:** The network structure of the Deep Residual U-Net to perform patch-wise segmentation along the coronal and sagittal axes.

Unit level	Conv layer	Filter	Stride	Output size
**Input**				$128 \times 64 \times 1$
Level 1	Conv 1 Conv 2	$3 \times 3/64$ $3 \times 3/64$	1	$128 \times 64 \times 64$ $128 \times 64 \times 64$
			1	
Level 2	Conv 3 Conv 4	$3 \times 3/128$ $3 \times 3/128$	2	$64 \times 32 \times 128$ $64 \times 32 \times 128$
			1	
Level 3	Conv 5 Conv 6	$3 \times 3/256$ $3 \times 3/256$	2	$32 \times 16 \times 256$ $32 \times 16 \times 256$
			1	
Level 4	Conv 7 Conv 8	$3 \times 3/512$ $3 \times 3/512$	2	$16 \times 8 \times 512$ $16 \times 8 \times 512$
			1	
Level 5	Conv 9 Conv 10	$3 \times 3/256$ $3 \times 3/256$	1	$32 \times 16 \times 256$ $32 \times 16 \times 256$
			1	
Level 6	Conv 11 Conv 12	$3 \times 3/128$ $3 \times 3/128$	1	$64 \times 32 \times 128$ $64 \times 32 \times 128$
			1	
Level 7	Conv 13 Conv 14	$3 \times 3/64$ $3 \times 3/64$	1	$128 \times 64 \times 64$ $128 \times 64 \times 64$
			1	
**Output**	Conv 15	$1 \times 1$	1	$128 \times 64 \times 1$

#### Reconstruction of the nodule shape

After obtaining the nodule segmentation for each plane, we apply the consensus module to calculate the final segmentation. The value of $k_{th}$ consensus pixel $c_k$ of segmentation mask is calculated as

4 $$\begin{aligned} c_k = \Gamma \left[ \sum _{i=0}^{M-1} S_{k_i} \right] , where\,k\in [0,N-1] \end{aligned}$$

where *S* represents the estimated segmentation, *M* is the number of estimations (three in our case, axial, coronal, and sagittal), *N* is the number of voxels in the VOI, and $\Gamma$ is defined as

5 $$\begin{aligned} \Gamma (g) = {\left\{ \begin{array}{ll} 1, &{} if\, g \ge (M*C_R) \\ 0, &{} Otherwise \end{array}\right. } \end{aligned}$$

Here, $C_R$ is the consensus ratio set as 0.5 to represent a 50% consensus.

## Experimental setup

### Dataset and pre-processing

All the experiments were carried out in accordance with relevant guidelines. We acknowledge the National Cancer Institute and the Foundation for the National Institutes of Health, and their critical role in the creation of the free publicly available database; the Lung Image Database Consortium and Image Database Resource Initiative (LIDC–IDRI) Database^12,31^ used in this study is freely available to browse, download, and use for commercial, scientific and educational purposes as outlined in the Creative Commons Attribution 3.0 unported License.

The LIDC–IDRI is the largest repository of CT scans to facilitate computer-aided systems on the assessment of lung nodule detection, classification and quantification. This dataset is composed of 1,018 cases of diagnostic and lung cancer screening thoracic CT scans with marked-up annotated lesions belonging to 1,010 patients. Each subject in the dataset includes images from a clinical thoracic CT scan and the results of a two-phase image annotation process performed by four experienced thoracic radiologists. We consider the nodules that are annotated by all the four radiologists and have a diameter of no less than 3 mm, as in used previous studies^32,33^. Because of the inter-variability among the four radiologists, a 50% consensus criterion^34^ is opted to generate the ground truth boundary of the pulmonary nodule segmentation and a python library named pyLIDC is used for this purpose. From LIDC dataset, a total of 893 nodules are selected and randomly divided into training, validation, and testing sets consisting on 356 (40%), 45 (5%), and 492 (55%) of nodules, respectively.

LIDC dataset contains scans acquired from various facilities and scanners. Therefore, it comes with the range of pixel spacing and slice thickness. These parameters play a vital role in nodule appearance. Especially, slice thickness greatly affects the coronal and sagittal views of nodule. In most of the LIDC scans, slice thickness is greater than pixel spacing, ranges from 0.45 to 5.0 mm. Therefore, slice thickness of each scan has been normalized to its corresponding pixel spacing to improve the visualization of nodule along coronal and sagittal view. For instance, if a scan has pixel spacing and slice thickness of $0.66\,\hbox {mm} \times 0.66\,\hbox {mm}$ and 2.5 mm, respectively. Then, the slice thickness is normalized to pixel spacing (i.e., 0.66 mm). However, the pixel spacing has kept intact, since it is was less than one for all the scans, which results good quality axial view of nodules.

The intensities of DICOM images are also normalized, from 0 to 1, by leveraging the DICOM tags information about window center (WC) and window width (WW). It can be defined as:

6 $$\begin{aligned} {I_{normalized} = \frac{I-Min}{Max - Min},} \end{aligned}$$

where $Min=WC-WW/2$, $Max = WC + WW/2$, *I* represents the original and $I_{normalized}$ denotes the normalized image.

Unlike previous studies^8,25–27^, those used a fixed margin strategy while extracting the ROIs for training purpose, we use random margins strategy. In the fixed margin approach, the margins shown in Fig. 4 are kept constant and of same size, subsequently nodule always appears at the centre of the ROI, which refrains the model learning from the possibility of nodules existence at the corners of ROI. However, in our solution, it is crucial to detect the nodule presence anywhere within a given ROI, since the A-ROI algorithm calculates the ROI for next slice by exploiting the nodule position in the current ROI. The networks are enabled to detect the nodule at any position within the ROI, by preparing the training data with random margins strategy. The values of all four margins are generated with a random function; restricted between zero and the maximum diameter of a nodule within the slice.

For patch-wise investigations of a nodule, the network should also learn about the absence of nodule in a given ROI to accurately determine the VOI, so we also include multiple non-nodule-containing ROIs from both sides (i.e., after the top and bottom slices) of the nodule. Since, nodule consists of several number of slices (as shown in Fig. 5), depending upon the nodule size, and we extract the ROI from each slice, thus, we get multiple ROIs from single nodule. Therefore, from 356 nodules, which are used for training, a total of 12,821 ROIs (images) are created and are utilized for training purpose.

### Implementation details

**Figure 7:**
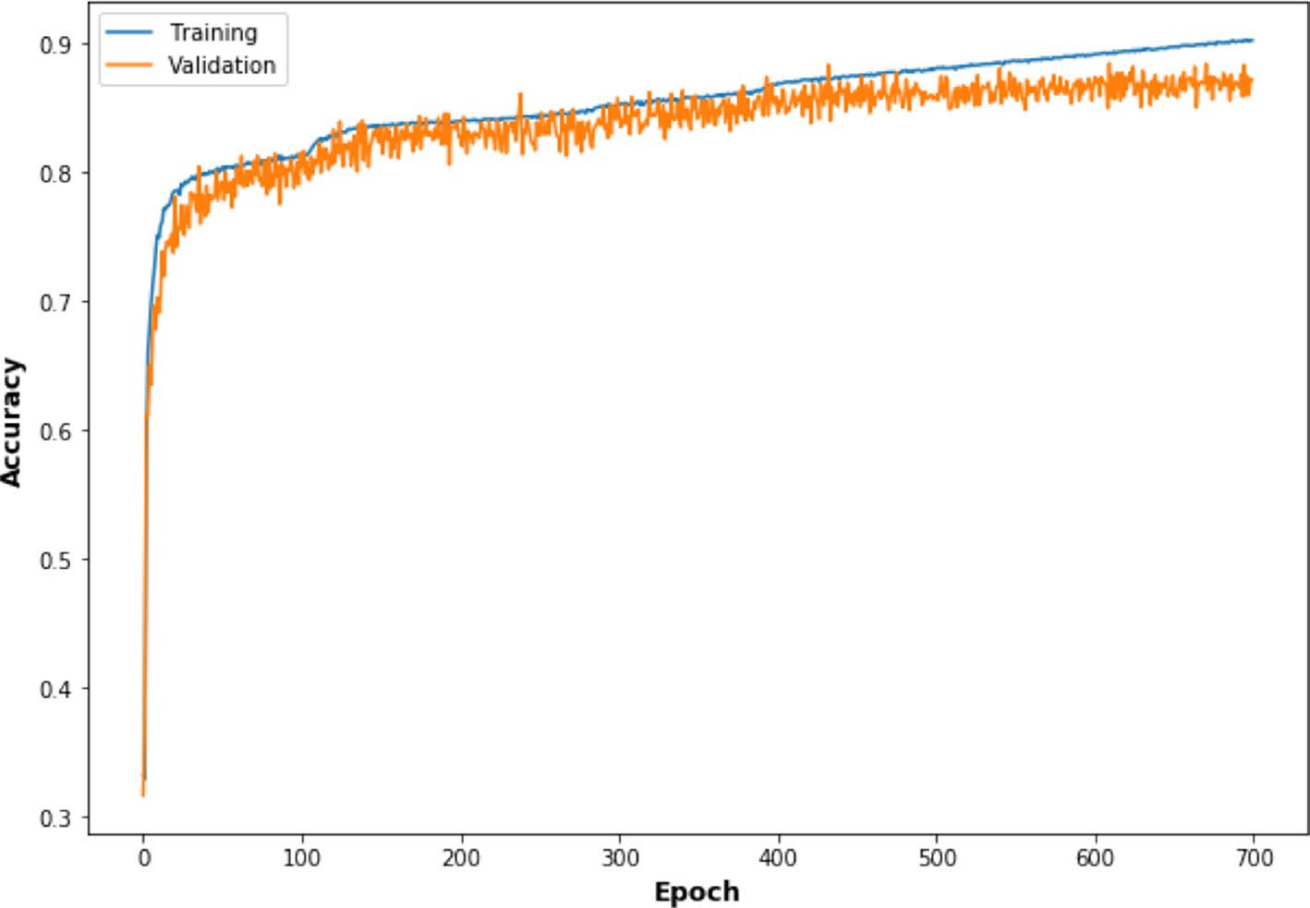
The graph of training and validation accuracy vs. the number of training epochs of Res-UNet.

The proposed model was implemented using Keras^35^ framework and optimized by minimizing Eq. (3) through SGD algorithm, and trained on 12,821 images sized $128\times 128$. We begin training the model with random weights and with learning rate of $10^{-4}$. We use mini-batch size of 8 on a NVIDIA TESLA V100 TENSOR CORE GPU. The network converges within 700 epochs and it takes 4 h and 51 min to complete the training. The learning curve of the network has been shown in Fig. 7.

### Evaluation parameters

We use following evaluation parameters to evaluate the perform of our proposed method.

#### Dice similarity coefficient

Equation (7) defines the dice similarity coefficient (DSC), which is widely used parameter to evaluate the degree of overlap of predicted segment ($S_{Pred}$) with reference segment ($S_{Ref}$)^8,18^. The DSC values ranges [0,1], while 0 and 1 indicate no overlap and complete overlap, respectively.

7 $$\begin{aligned} DSC = \frac{2*S_{Pred}\cap S_{Ref}}{S_{Pred} \, {\cup } \, S_{Ref}} \end{aligned}$$

#### Sensitivity and positive predictive value

The pixel classification performance and correctness of the segmentation area are measured by the sensitivity (SEN) and the positive predictive value (PPV), which are defined as

8 $$\begin{aligned} SEN= & {} \frac{S_{Pred}\cap S_{Ref}}{S_{Ref}} \end{aligned}$$

9 $$\begin{aligned} PPV= & {} \frac{S_{Pred}\cap S_{Ref}}{S_{Pred}} \end{aligned}$$

#### Hausdorff distance

Hausdorff distance (HD)^36^ is another commonly used metric for the evaluation of medical segmentation^37^, which measures the dissimilarity between two sets of points. The directed Hausdoff distance (H) between two point sets $S_{Ref}$ and $S_{Pred}$ is the maximum distance between each point $x \in S_{Ref}$ to its nearest neighbour $y \in S_{Pred}$. That is

10 $$\begin{aligned} {H(S_{Ref},S_{Pred}) = max_{x\in S_{Ref}}\{min_{y\in S_{Pred}}\{ \Vert x,y \Vert \}\},} \end{aligned}$$

where $\Vert \cdot ,\cdot \Vert$ is any norm i.e., the euclidean distance function. Note that $H(S_{Ref},S_{Pred}) \ne H(S_{Pred},S_{Ref})$ and thus the directed Hausdorff distance is not symmetric. The Hausdorff distance *HD* is the maximum of the directed Hausdorff distances in both directions and thus it is symmetric. *HD* is given by:

11 $$\begin{aligned} {HD(S_{Ref},S_{Pred}) = max\{H(S_{Ref},S_{Pred}) , H(S_{Pred},S_{Ref})\}.} \end{aligned}$$

## Results and discussion

We performed various experiments on the LIDC-IDRI dataset to evaluate the performance of our approach . The results were analyzed based on aspects of the effect of the $R_T$ value, overall performance, robustness, and visual performance. The following subsections review our analysis.

### Performance sensitivity to the $R_T$ value

**Figure 8:**
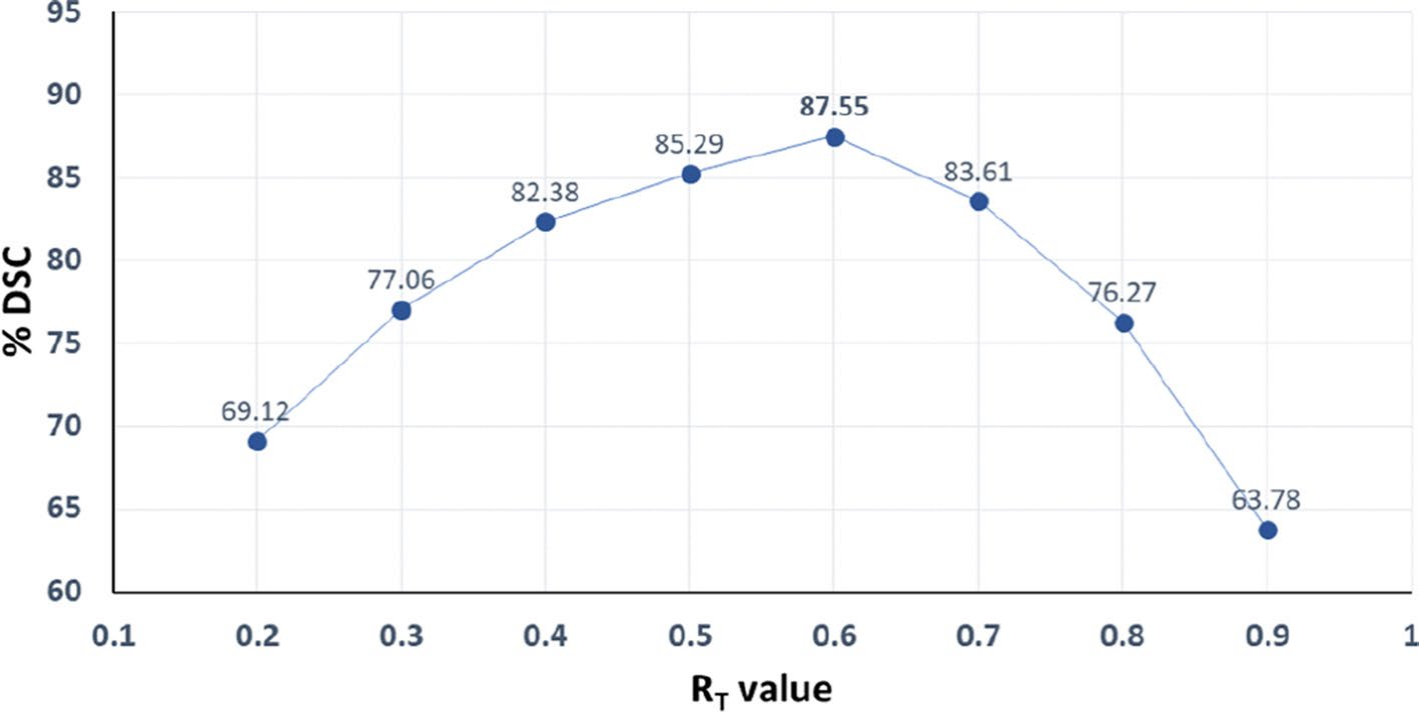
The graph between various values of $R_T$ and the corresponding performances in term of % DSC on the validation data.

The value of $R_T$ is determined experimentally by using validation data; multiple experiments were performed with various values of $R_T$ to obtain its optimum value. Figure 8 shows a range of $R_T$ values and their respective overall performance. With an increase in the value of $R_T$, the performance also increases until it reaches a specific point (i.e., 0.6), after which the performance decreases. The graph illustrates that for lower $R_T$ representing a larger ROI, the performance is degraded because the ROI is not focused on a single nodule and includes redundant information or non-nodule structures. This extraneous information makes the segmentation process challenging, consequently reducing overall performance. On the other hand, when a high value of $R_T$ is selected, the ROI not only focuses on a nodule but also remains very close to the nodule boundary, which is insufficient to handle small displacements of the nodule across adjacent slices. Therefore, a moderate value of $R_T$ provides the best performance, and we used the same value of 0.6 for $R_T$ in all experiments reported.

### Overall performance

The overall performance of the proposed method is evaluated by using all evaluation parameters mentioned above. Table 3 lists these results for various methods, and the adaptive ROI method significantly outperforms previous state-of-the-art techniques. To demonstrate the effectiveness of adaptive ROI, we also applied a constant ROI with Multi-view Residual leaning, which also outperformed the existing techniques reflecting the power of residual learning, as it has not been previously used for nodule segmentation.

**Table 3:** The mean ± standard deviation for quantitative results of various segmentation methods with the best performance indicated in bold.

Methodology	DSC (%)	SEN (%)	PPV (%)
Central focused CNN^8^	78.55 ± 12.49	86.01 ± 15.22	75.79 ± 14.73
Multi-crop CNN^38^	77.51 ± 11.40	88.83 ± 12.34	71.42 ± 14.78
Multi-view CNN^39^	75.89 ± 12.99	87.16 ± 12.91	70.81 ± 17.57
Multi-view deep CNN^25^	77.85 ± 12.94	86.96 ± 15.73	77.33 ± 13.26
Multichannel ROI based on deep structured algorithms^40^	77.01 ± 12.93	85.45 ± 15.97	73.52 ± 14.62
Cascaded dual-pathway Res-Net^10^	81.58 ± 11.05	87.30 ± 14.30	79.71 ± 13.59
Unsupervised metaheuristic search^9^	82.34 ± 5.40	87.10 ± 9.78	85.59 ± 11.06
Constant ROI with multi-view deep residual learning	84.35 ± 11.72	89.02 ± 8.91	86.73 ± 10.11
A-ROI with multi-view deep residual learning	87.55 ± 10.58	91.62 ± 8.47	88.24 ± 9.52

The results in Table 3 also suggest that the incorporation of adaptive ROI significantly improves the results, with the average DCS value increasing by approximately 3%. The reason for such high performance is due to the advantage explained in Fig. 5. The approach reduces the size of the ROI enabling it to focus only on the nodule and eliminating possible redundant information (i.e., similar non-nodule structures) from the ROI, which subsequently assists the residual U-Net architecture for the classification of the nodule and non-nodule voxels. On the other hand, the constant ROI must cover a large area (i.e., surrounding of nodule) to incorporate the entire shape of the nodule, which impacts the performance of the network.

### Evaluation of robustness

The LIDC–IDRI dataset also provides annotations about the levels of nine characteristics of the nodules, such as Subtlety, Internal Structure, Calcification, Sphericity, Margin, Lobulation, Speculation, Texture, and Malignancy. The sphericity of nodules, the likelihood of malignancy, and other properties are represented by these characteristics^8^. We partitioned the test data into groups according to the level of each characteristic and extracted the results against each level, as presented in Table 4. The dice score achieved for each group is similar, indicating the robustness of our approach.

**Table 4:** Average DSC for different nodule types in the LIDC-IDRI testing set.

Characteristics	Characteristic scores					
	1	2	3	4	5	6
Calcification	_	_	84.61 [18]	83.88 [42]	86.24 [27]	88.15[405]
Internal structure	87.62 [487]	79.27 [3]	_	81.48 [2]	_	_
Lobulation	86.96 [201]	88.78 [164]	86.56 [78]	85.94 [31]	89.74 [18]	_
Malignancy	85.67 [39]	86.56 [114]	89.26 [163]	86.67 [98]	87.52 [78]	_
Margin	86.42 [9]	85.61 [37]	86.73 [78]	88.21 [232]	87.51 [136]	_
Sphericity	_	86.79 [38]	85.28 [153]	89.16 [218]	88.12 [83]	_
Speculation	89.29 [257]	85.18 [165]	86.96 [32]	86.37 [14]	86.68 [24]	_
Subtlety	80.52 [4]	82.65 [22]	87.51 [131]	87.09 [238]	90.17 [97]	_
Texture	82.18 [11]	85.67 [18]	86.54 [26]	87.55 [107]	87.93 [330]	_

### Visual analysis

**Figure 9:**
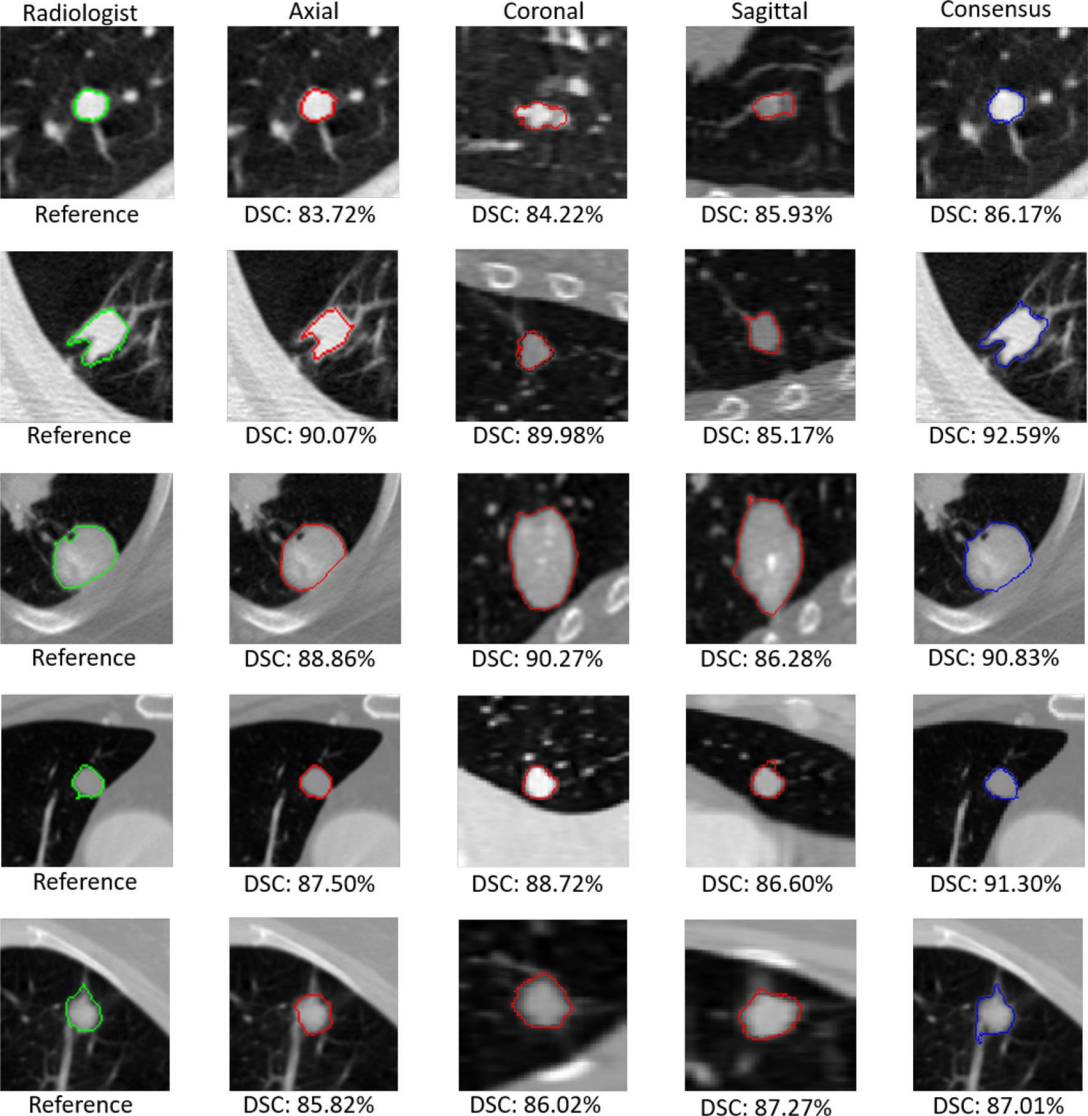
The segmentation results of proposed approach, at different stages (i.e., after employing segmentation along axial, coronal, sagittal axis) and finally the segmentation outputs of consensus module have been presented with their respective dice scores.

The results of our method are visualized to analyze its performance at various stages of the proposed approach. In Fig. 9, the segmentation outputs, following the axial, coronal, and sagittal view investigations, are shown with the output of the consensus module presented in the last column. Nodules of various types from test data are randomly selected, including the attached, non-attached, and nodule with extreme subtlety. The results suggest that adaptive ROI with residual learning enhances the performance of the axial view investigation. However, in the cases where the penetration of the nodule along the z-axis is higher than the presence of the nodule on the x–y plane, the performance along the sagittal and coronal axes is only slightly higher. Subsequently, in a few cases, the performance following the consensus module is also slightly reduced. However, according to the dice scores of the outputs following the axial, coronal, sagittal, and consensus modules listed in Table 5, the performance still improves significantly after applying the consensus module.

**Figure 10:**
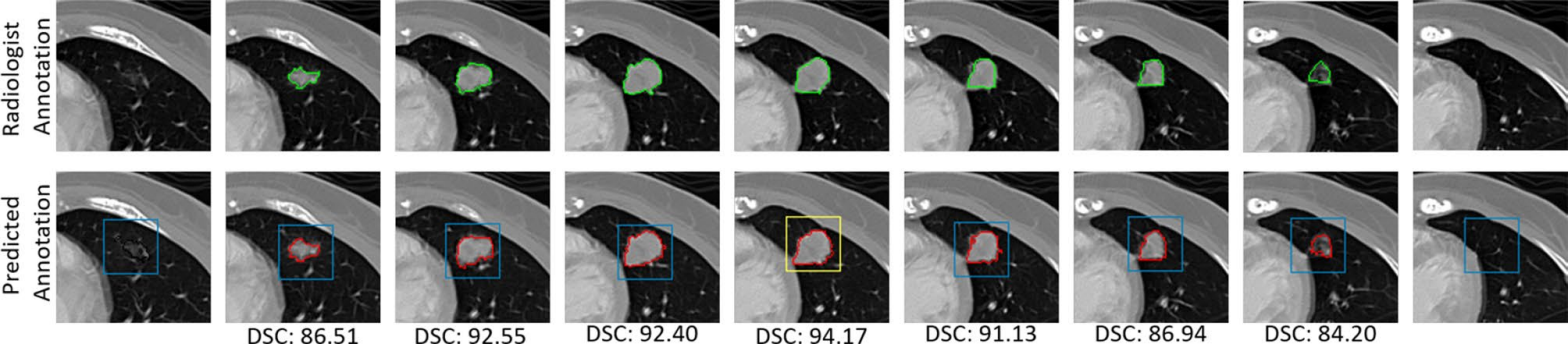
The segmentation performance of our proposed approach on each slice of a single nodule with the corresponding dice scores. From columns 1–9, the sequence of slices is observed with the nodule seen in columns 2–7 and no nodule in columns 1 and 9. The squares marked in the predicted segmentation row depict the ROIs, where yellow represents the initial (manually provided) ROI, and blue represents the adaptive ROIs.

**Table 5:** Average dice score and Hausdorff distance for each stage of the proposed approach.

	Axial	Coronal	Sagittal	Consensus
Dice score (%)	$85.29\pm 9.78$	$84.76\pm 12.45$	$83.58\pm 8.93$	$87.55\pm 10.58$
Hausdorff distance (mm)	$2.76\pm 1.68$	$4.78\pm 2.21$	$3.41\pm 1.94$	$2.93\pm 1.87$

Because our method automatically extracts the VOI, an estimation of nodule penetration in the surrounding slices must be evaluated. Figure 10 shows the visual results from a complete nodule. As described above, a manual ROI is needed to initialize the segmentation process, which is presented in yellow, and the adaptive ROIs are calculated through the A-ROI algorithm for the surrounding slices, as presented in blue. The detected presence of a nodule in each slice is highlighted with the corresponding dice scores. Our method successfully detects the penetration of the nodule in each slice and stops the investigation immediately after the end of the nodule is reached from both sides.

### Limitations of A-ROI algorithm

**Figure 11:**
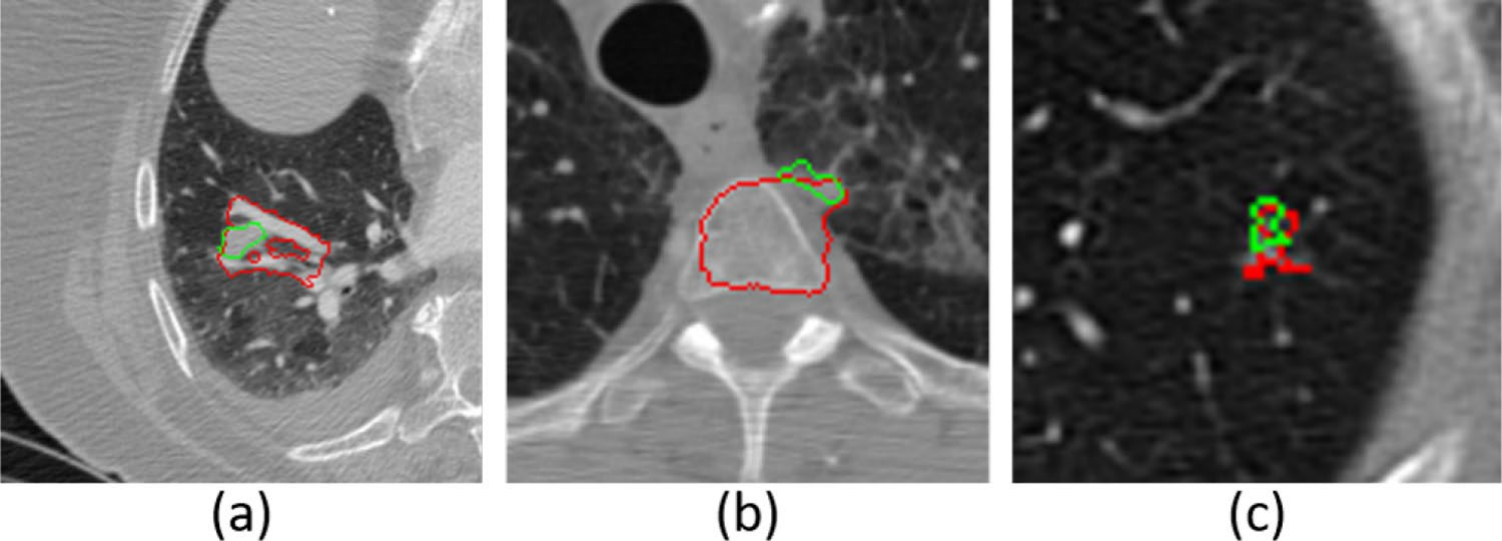
Visual examples of pulmonary nodule segmentation results on which the proposed method failed to accurately segment the nodule boundary.

Figure 11 shows representative challenging images where the proposed A-ROI algorithm could not accurately segment the pulmonary nodule. There are two main reasons of this failure. First, as it can be observed in Fig. 11a,b that nodules are largely connected with non-nodule structures in the lungs and nodules boundaries are extremely fuzzy from two sides. Therefore, during the patch-wise investigation of nodule, A-ROI algorithm fails to determine the optimum ROI for next slice, which subsequently results into compromised performance. This limitation can remedied by manually re-adjusting the ROI.

Secondly, in the cases where the nodule appearance along the axial axis is very small as compared to other axes as shown in Fig. 11c, A-ROI algorithm suffers to accurately detect the boundary of nodule because of higher re-scaling size (i.e., $128\times 128$). For instance, the nodule size in axial slice view is 5 $\times$ 5 and according to A-ROI algorithm the optimum size of ROI is 9 $\times$ 9, which is further resized to $128\times 128$ to fed into Res-UNet for segmentation. Such huge difference between the actual size and the re-scaling size distort the resultant image and network fails to accurately segment the nodule. This limitation can be overcome by training multiple networks with different re-scaling dimensions (i.e., $64\times 64$, $32\times 32$, and $16\times 16$) so radiologist may opt according to the nodule size.

## Conclusion

We introduced a novel, two-stage pulmonary nodule segmentation technique that generates a highly accurate, 3-D segmentation of a nodule with minimum human interaction. First, the technique requires a 2-D ROI along the axial axis containing the nodule as input and then automatically estimates the volume of interest (VOI). For VOI extraction, we proposed the novel adaptive ROI algorithm with a Deep Residual U-Net architecture to leverage the position of the nodule within the ROI of the current slice to adjust the position and shape of the ROI for the next slice. Second, additional patch-wise segmentations of the nodule along the coronal and sagittal axes are performed by applying two Residual U-Nets. Finally, the segmentation outputs of the axial, coronal, and sagittal axes are processed through the consensus module to generate the final segmentation mask. Our technique was evaluated on the LIDC-IDRI dataset with quantitative and visual results. We demonstrated that our approach outperforms the previous state-of-the-art techniques in terms of segmentation dice scores and is significantly robust for various nodule types, suggesting its suitability for real-time implementation to minimize the radiologist effort through the automatic estimation of the VOI and to enhance the accuracy of segmentation. Future research will include an extension of the framework to perform a time-based analysis of a selected nodule by using sequential scans of a patient, which will assist radiologists in estimating the malignancy of an identified nodule by observing its development over time.
